# Comparative Outcomes of Robot-Assisted and Laparoscopic Pyeloplasty in Infants and Toddlers: A Systematic Review and Meta-Analysis

**DOI:** 10.3390/children13060728

**Published:** 2026-05-23

**Authors:** Maciej Szyduczyński, Johannes Korneliussen, Adrianna Jażdżewska, Daria Sosińska, Ewelina Wojciechowska, Stefan Anzelewicz, Andrzej Gołębiewski

**Affiliations:** 1 Department of Surgery and Urology for Children and Adolescents, Faculty of Medicine, Medical University of Gdańsk, Smoluchowskiego 17, 80-214 Gdańsk, Poland; 2 Faculty of Medicine, Medical University of Gdańsk, Smoluchowskiego 17, 80-214 Gdańsk, Poland

**Keywords:** children, infants, pyeloplasty, laparoscopic, robotic, hydronephrosis

## Abstract

**Highlights:**

**What are the main findings?**

- Reduced length of stay—robotic-assisted laparoscopic pyeloplasty is associated with significantly shorter hospitalizations for infants and toddlers compared to conventional laparoscopy.
- Robotic surgery achieves these faster recovery times without compromising the clinical successes or safety of the procedure.

**What are the implications of the main findings?**

- These results advocate for including a robotic-assisted technique as an efficient surgical option for treating pediatric ureteropelvic junction obstruction.
- High-quality randomized trials are required to confirm these observations.

**Abstract:**

**Background**: Ureteropelvic junction obstruction (UPJO) represents a common congenital anomaly in infants and young children. While minimally invasive approaches, including laparoscopic pyeloplasty (LP) and robot-assisted laparoscopic pyeloplasty (RALP), have gained acceptance, comparative outcomes in children younger than 3 years have still not been well-established. This study aimed to evaluate the safety and efficacy of RALP versus LP in infants and children younger than 3 years or weighing < 15 kg. **Methods**: A systematic literature search of PubMed, Web of Science, and Embase was conducted through May 2025. Five retrospective cohort studies comprising 272 patients met inclusion criteria (age younger than 3 years or weighing < 15 kg). Meta-analysis was performed using random-effects models. **Results**: Hospital stay was significantly shorter in the RALP group compared with LP (mean difference = −1.69 days; 95% CI: −2.71 to −0.67). No statistically significant differences were observed in operative time, complication rates, or success rates between approaches. **Conclusions**: RALP is associated with significantly reduced hospitalization time compared with LP in infants and young children, while maintaining comparable safety and efficacy profiles. These findings support RALP as a valuable minimally invasive option in this challenging patient population, though larger prospective studies are warranted.

## 1. Introduction

### 1.1. Clinical Background and Epidemiology

Ureteropelvic junction obstruction (UPJO) represents the most frequent cause of congenital hydronephrosis, typically detected on prenatal ultrasonography. With an incidence of approximately 1 in 1500 newborns and a 2:1 male predominance, UPJO may progress to renal impairment if left untreated [1,2].

### 1.2. Diagnostic Challenges

The diagnostic paradigm for UPJO remains challenging, as no universal threshold distinguishes true obstruction from non-obstructive dilatation. Current definitions emphasize any restriction to urinary outflow potentially compromising renal function [3]. Serial monitoring with consistent methodology at specialized centers provides the most reliable assessment [4], with treatment decisions integrating clinical presentation, imaging findings, and renal functional parameters [5]. The main goal is to prevent long-term kidney damage by identifying and treating significant obstruction early [5].

### 1.3. Treatment Options

Management strategies must integrate clinical presentation, imaging findings, and renal functional parameters. Conservative management may be considered in selected cases of unilateral UPJO in infants under two years of age, although current evidence is limited by methodological bias and heterogeneity across studies [6]. Surgical intervention is indicated for symptomatic obstruction, recurrent infections, or declining renal function [1].

### 1.4. Evolution of Surgical Management

Open dismembered pyeloplasty (Hynes–Anderson technique) remains the reference standard, offering durable outcomes. Minimally invasive techniques, including laparoscopic pyeloplasty (LP), retroperitoneoscopic pyeloplasty, and robot-assisted laparoscopic pyeloplasty (RALP), have demonstrated comparable efficacy in experienced centers [7,8]. Nevertheless, robust comparative data in infants and young children remain scarce. To address this gap, we conducted a meta-analysis comparing outcomes of RALP and LP in pediatric UPJO.

### 1.5. Rationale for Current Study

While several systematic reviews have compared RALP and LP in pediatric populations, most have included heterogeneous age groups with limited focus on infants and very small children. The distinct anatomical considerations and technical challenges inherent to this population necessitate dedicated analysis to guide clinical decision-making.

Previous meta-analyses have yielded conflicting results on the comparative effectiveness of RALP versus LP due to variations in outcome definitions, follow-up duration, and patient selection criteria. Furthermore, the rapid evolution of robotic technology and the increasing experience of surgeons necessitate an updated analysis of contemporary outcomes [9].

### 1.6. Study Objectives and Hypotheses

The primary objective of this systematic review and meta-analysis was to compare the safety and efficacy of RALP versus LP in infants and children younger than 3 years or weighing < 15 kg. We hypothesized that while both approaches would demonstrate high success rates, RALP might offer advantages in terms of operative efficiency and perioperative outcomes due to enhanced visualization and instrument dexterity, particularly relevant in the confined working spaces encountered in infant surgery. We also aimed to assess the quality of available evidence and identify priorities for future research in this specialized population.

## 2. Materials and Methods

### 2.1. Search Strategy

A comprehensive literature search was conducted across PubMed, Web of Science, and Embase. Studies published in any language were considered in the search, covering the period from each database’s inception up to 13 May 2025, in accordance with the predefined inclusion criteria. The following keywords were used in the search strategy: ((pyeloplasty OR “pelviureteric junction obstruction” OR “ureteropelvic junction obstruction” OR UPJO) AND (laparoscopic OR laparoscopy) AND (“robot-assisted” OR robotic OR RALP) AND (infant OR children OR pediatric OR child OR “under 3 years” OR “less than 3 years” OR “younger than 3 years”) AND (outcome OR “success rate” OR complications OR “operative time” OR “hospital stay” OR conversion OR reoperation OR recurrence)). This study protocol was registered through PROSPERO (registration no. CRD420251073297; https://www.crd.york.ac.uk/PROSPERO/ (accessed on 25 March 2026)).

### 2.2. Study Selection

A systematic review was performed following the Preferred Reporting Items for Systematic Reviews and Meta-Analyses (PRISMA) guidelines throughout the research process. Two investigators independently screened titles, abstracts, and full-text articles. The inclusion criteria comprised (1) comparative studies of RALP versus LP; (2) participants younger than 3 years or weighing < 15 kg; (3) reported perioperative outcomes; and (4) a minimum sample size of 10 patients. Discrepancies were resolved through consensus or third-author consultation.

To determine eligibility for each synthesis, we tabulated the intervention characteristics, patient ages and outcome measures. These were then compared against our predefined criteria. Only studies that directly compared RALP and LP in children younger than 3 years or weighing < 15 kg and those that reported extractable perioperative outcomes were included in the data synthesis. The tabulation and comparison were performed in Microsoft Excel.

### 2.3. Data Extraction and Quality Assessment

The quality of the included studies was assessed using the modified Newcastle–Ottawa Scale (NOS), which evaluates three domains: selection, comparability, and outcome. Studies could receive up to 4 points for selection, 2 points for comparability, and 3 points for outcome, with a maximum total score of 9. A total score of 7 or higher indicated high-quality studies, scores between 4 and 6 reflected moderate quality, and scores of 3 or below indicated low-quality studies [10]. Two reviewers independently conducted quality assessment.

The certainty of evidence for each outcome was evaluated using the GRADE (Grading of Recommendations Assessment, Development, and Evaluation) approach [11]. Evidence from observational studies was initially categorized as low-certainty and subsequently assessed for downgrading based on five domains: risk of bias (informed by the NOS), inconsistency, indirectness, imprecision, and publication bias. The criteria for potential upgrading, such as large magnitude of effect, were also applied where appropriate. The assessment was performed using the GRADEpro Guideline Development Tool (GDT), resulting in four certainty levels: high, moderate, low, or very low.

Data extraction was performed using a standardized form, capturing demographic variables, operative parameters, ureteral stent placement, drain or nephrostomy tube placement, intraoperative cystostomy, complication rate, conversion rate, hospitalization time, success rate, follow-up time, and amounts of used analgesics.

### 2.4. Statistical Analysis

For statistical analysis, both dichotomous and continuous outcomes were pooled to estimate overall effect sizes, reported as odds ratios (ORs) for dichotomous data and mean differences (MDs) for continuous data, each with corresponding 95% confidence intervals (CIs) to compare outcomes between LP and RALP. The Mantel–Haenszel method was used for dichotomous outcomes, and the Inverse Variance method was used for continuous outcomes. A random-effects model was employed throughout, and heterogeneity was assessed using the I2 statistic. Leave-one-out sensitivity analysis was performed for each outcome in order to assess the robustness of the pooled estimates and to identify potential sources of statistical heterogeneity. When the data was reported as medians and ranges, the method described by Hozo et al. was used to estimate the means and standard deviation [12]. For data reported as medians and interquartile intervals (IQRs), we applied the method proposed by Wan et al. to derive corresponding mean and standard deviation values [13]. A *p*-value of <0.05 was considered statistically significant. All analyses were conducted using Review Manager version 5.4.1 (Cochrane IMS).

To address the clinical heterogeneity regarding patient age and to explore the impact of the age–weight spectrum on surgical outcomes, a subgroup analysis was performed. The included studies were categorized into two groups based on the population characteristics: the “infants only” subgroup, comprising studies that exclusively included patients younger than 12 months, and the “toddlers and infants” subgroup, comprising studies that included both toddlers and infants. It should be noted that the subgroup analyses were exploratory and inherently underpowered due to the limited number of studies, and thus their results should be interpreted with caution.

## 3. Results

### 3.1. Search Results and Characteristics of Included Studies

**Figure 1:**
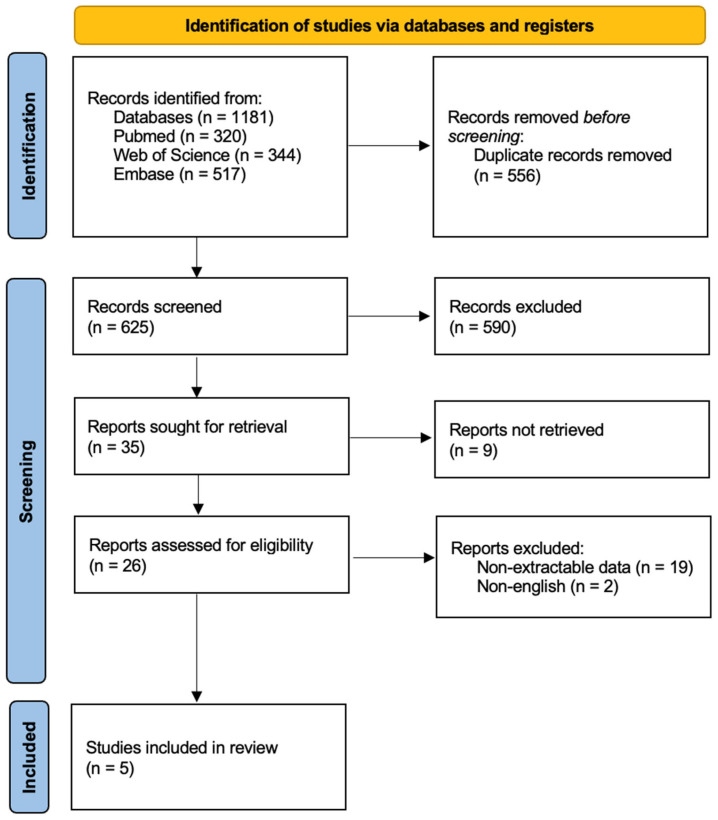
PRISMA flow diagram.

The systematic literature search identified 625 unique records, of which five retrospective cohort studies met the inclusion criteria (total *n* = 272 patients) (Figure 1). All studies were rated as high-quality (NOS ≥ 7). RALP was performed in 148 patients (54.4%) and LP in 124 patients (45.6%), with an overall male predominance (67.8%). The study characteristics are summarized in Table 1.

### 3.2. Certainty of Evidence and Publication Bias Assessment

The certainty of evidence for all evaluated outcomes—including success rates, complication rates, operative times, and times of hospitalization—was assessed as very low according to the GRADE criteria. Detailed results are presented in the Summary of Findings table (Table 2).

Despite the high methodological quality of the included studies, the evidence was primarily downgraded due to (1) very serious inconsistency in operative time and hospitalization length and (2) serious imprecision for operative time and success and complication rates, characterized by small sample sizes and 95% confidence intervals crossing the line-of-null effect.

Publication bias could not be meaningfully assessed because of the small number of included studies.

### 3.3. Differences Between LP and RALP

The total number of patients included in this analysis was 272. RALP was performed in 54.4% of all cases (148 patients) and LP in 45.6% (124 cases). The male-to-female ratio was 2:1, with males accounting for 67.8% (162) and females for 33.2% (77) of all cases.

The youngest patients undergoing RALP and LP were, respectively, 1.4 months and 3 months old at the time of the operation [14]. The lowest body masses recorded at the time of surgery were 4.7 kg and 6 kg for RALP and LP, respectively [14].

**Figure 2:**
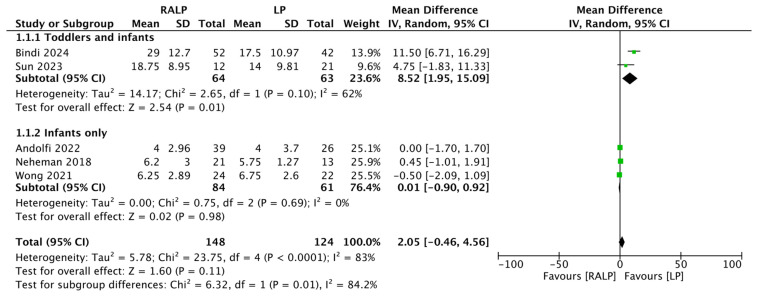
Forest plot comparing the ages of the patients between the groups [14,15,16,17,18].

All authors reported the ages of the patients [14,15,16,17,18]. The mean age at surgery was 14.66 ± 13.88 months in the RALP group and 10.94 ± 9.61 months in the LP group, with no statistically significant difference between the groups (MD = 2.05; *p* = 0.11) (Figure 2). However, a subgroup analysis focusing on the combined “toddlers and infants” group revealed that the patients in the RALP group were older (MD = 8.52; *p* = 0.01). No such difference was observed in the infants-only subgroup. Leave-one-out analysis showed that the study by Bindi et al. was the main source of heterogeneity in this analysis [18]. After the removal of that study, the heterogeneity decreased to 0%, yet the statistical insignificance remained unchanged (MD = 0.1, *p* = 0.83) (Supplementary Figure S1).

**Figure 3:**
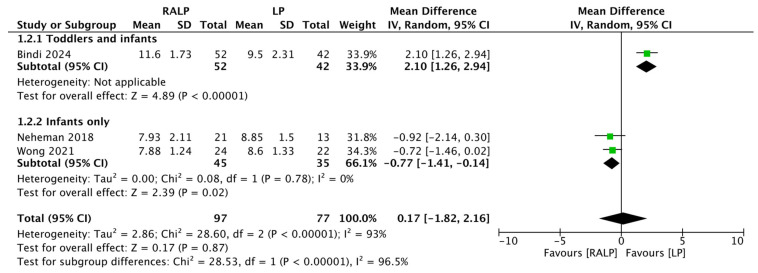
Forest plot comparing the weights of patients in both groups [14,16,18].

Three authors reported patients’ body masses during surgery [14,16,18]. The mean weights of patients undergoing surgery were 9.89 ± 2.51 kg and 9.13 ± 1.97 kg for RALP and LP, respectively. While the difference between the two groups was insignificant (MD = 0.17; *p* = 0.87), subgroup analysis revealed a different trend (Figure 3). In the infants-only subgroup, patients undergoing RALP had significantly lower body masses compared with the LP group (MD = −0.77; *p* = 0.02). Leave-one-out analysis showed that study by Bindi et al. was the main source of heterogeneity in this analysis. After the removal of that study, heterogeneity decreased to 0% and the results were statistically significant (MD = −0.77, *p* = 0.02) (Supplementary Figure S1).

**Figure 4:**
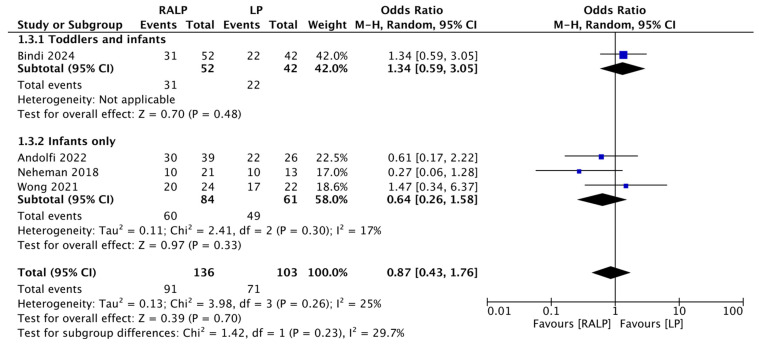
Forest plot comparing the sexes of the patients between the groups [14,15,16,18].

The sexes of patients have been reported by four authors, with no difference between RALP patients (66.9% males) and LP patients (68.9% males): OR = 0.87; *p* = 0.70 [14,15,16,18] (Figure 4).

**Figure 5:**
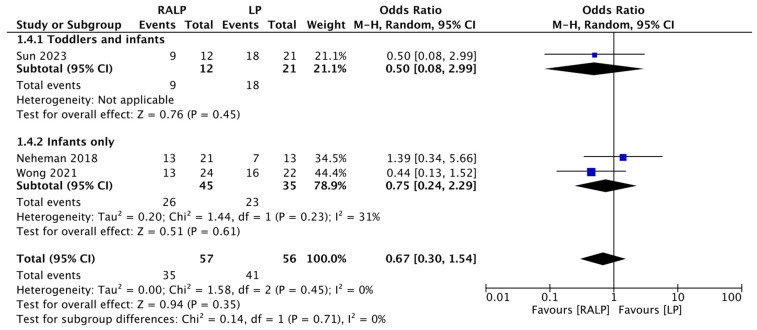
Forest plot comparing the sides of the operated kidneys between the groups [14,15,17].

The sides of the kidneys undergoing surgery have been reported in three publications [14,15,17]. There was no difference between the RALP group (61.4% left-sided) and LP group (73.2% left-sided): OR = 0.67; *p* = 0.35 (Figure 5).

**Figure 6:**
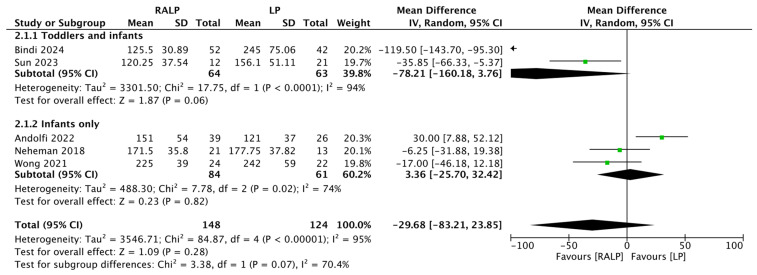
Forest plot comparing operative time between the groups [14,15,16,17,18].

Mean operative time was reported in all five analyzed studies [14,15,16,17,18]. The duration of RALP was 146.35 ± 64.50 min, and it was 196.62 ± 77.68 min for LP. There was, however, no statistical significance in these results (MD = −29.68; *p* = 0.28) (Figure 6). The initial analysis showed high heterogeneity (95%). The leave-one-out test showed that after removal of the study by Bindi et al. [18], the heterogeneity remained at a high level of 78%, yet preserving the outcome insignificance (MD = 6.04; *p* = 0.68) (Supplementary Figure S1). This implicates that the heterogeneity in this outcome may have originated from systemic differences between pediatric centers and the varying experience of surgeons rather than from a single outlier study.

**Figure 7:**
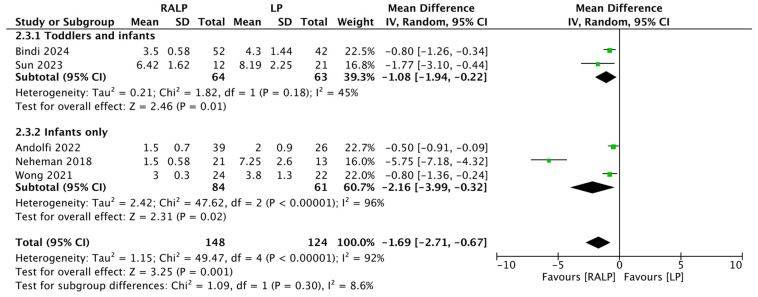
Forest plot comparing time of hospitalization between the groups [14,15,16,17,18].

All authors reported time of hospitalization [14,15,16,17,18]. It was statistically significant, with 2.84 ± 1.56 days for patients undergoing RALP and 4.7 ± 2.67 days for patients undergoing LP (MD = −1.69; *p* = 0.001) (Figure 7). The test for subgroup differences revealed no significant inconsistency, indicating that the clinical benefit of RALP may be comparable between infants and older children within this population. Although the initial heterogeneity was high, the leave-one-out sensitivity analysis identified the study by Neheman et al. [14] as the primary contributor to the statistical heterogeneity (Supplementary Figure S1). After its exclusion, the heterogeneity markedly decreased to 19%, while the significance of the results remained highly robust (MD = −0.73, *p* < 0.0001). This confirms that the reduction in postoperative hospital stay is a consistent benefit of the robotic approach regardless of the individual variations in institutional discharge protocols.

**Figure 8:**
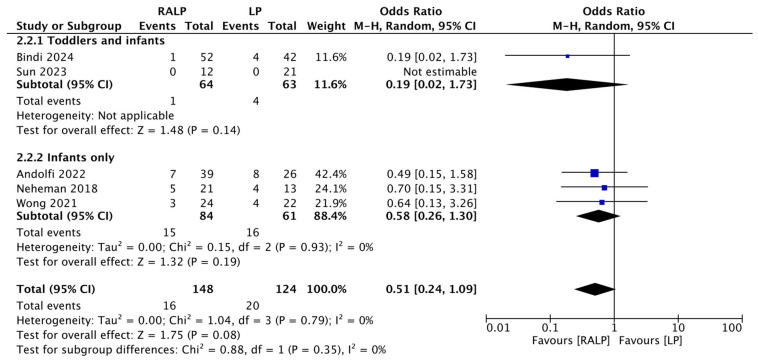
Forest plot comparing postoperative complication rates between the groups [14,15,16,17,18].

Postoperative complications were reported by all authors [14,15,16,17,18]. All studies reported a lower or equal complication rate in the RALP group (10.8%) compared with the LP group (16.1%), although the result did not reach statistical significance (OR = 0.51; *p* = 0.08) (Figure 8).

**Figure 9:**
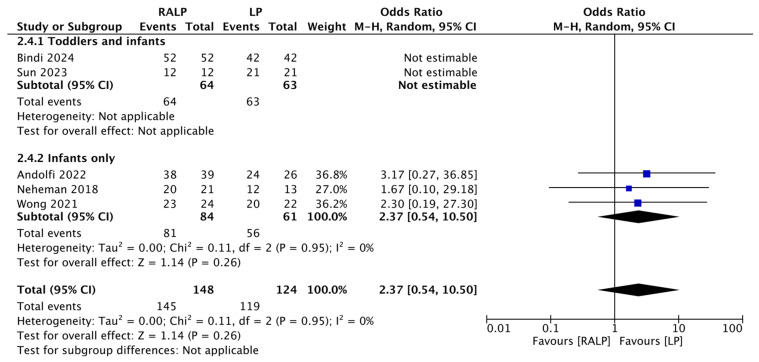
Forest plot comparing the success rates between the groups [14,15,16,17,18].

While all five authors reported the success rates, they were similar in both groups in two of the studies [14,15,16,17,18]. The success rates were 98% and 95.6% for the RALP and LP groups, respectively, failing to reach statistical significance (OR = 2.37; *p* = 0.26) (Figure 9).

## 4. Discussion

Over the past two decades, minimally invasive approaches, including LP and RALP, have gained prominence in pediatric urology, offering reduced postoperative pain, shorter hospital stays, and improved cosmetic results while maintaining comparable success rates to open surgery [19,20,21]. RALP in children of less than 6 months of age was first reported by Kutikov et al. in 2006 [22]. These technological advances continue to shape the management and prognosis of UPJO in the pediatric population.

The present study aimed to assess outcomes of RALP and LP in infants and small children younger than 3 years or weighing < 15 kg. Our findings present that hospital stays were significantly shorter in the RALP group, whereas operative time, complication rates, and success rates were comparable between the two techniques.

These results are consistent with prior evidence. Cascini et al., in a meta-analysis of 3145 infants, demonstrated shorter hospitalization for minimally invasive surgery (MIS). However, no significant differences were observed in complication rates or surgical success between open, laparoscopic, and robotic approaches [8]. Similarly, Chan et al., in a large multicenter cohort, found a shorter length of stay for RALP compared to open pyeloplasty (OP). However, this was statistically significant in patients of all ages in total and not exclusively in the younger age group [23].

Several meta-analyses have supported the superiority of RALP in terms of hospitalization time and postoperative recovery. Chen et al., analyzing over 3000 pediatric patients of all ages, reported significantly shorter operative time and hospitalization and lower postoperative complication rates in the RALP group compared with LP [24]. Our data align partially with these findings, particularly in regard to hospitalization, though we did not confirm differences in surgical success or complication rates, possibly due to smaller pooled numbers and stricter inclusion criteria focused on younger children.

Notably, Hu et al. and Ganpule et al. also found shorter hospitalization following RALP in young children and infants, further validating our conclusions [25,26]. On the other hand, other studies, such as those by Esposito et al. and Ebert et al., showed no statistically significant differences in complication rates, surgical duration, or hospital stay between the two approaches, highlighting the variability across different centers and patient populations [27,28].

It is hypothesized that in children younger than 3 years, the extremely confined abdominal workspace strongly restricts the excursion of conventional laparoscopic instruments, leading to fulcrum conflicts and limited triangulation. The robotic platform overcomes these limitations through instruments with seven degrees of freedom and a “wristed” articulation combined with high-definition 3-D magnification and tremor filtration, allowing extreme precision [29]. This increased dexterity translates directly into reduced collateral tissue trauma and a weaker surgical stress response: gentler dissection and minimal thermal spread reduce the release of pro-inflammatory cytokines. This particularly benefits the youngest children, whose immature immune systems are prone to an exaggerated systemic reaction [30,31]. Moreover, the steady, magnified view and precise tissue handling limited excessive traction on the bowel, reducing postoperative ileus and lowering pain intensity. This, in turn, decreased opioid requirements—which themselves prolong gut dysmotility—and enabled earlier enteral feeding and mobilization, ultimately shortening the hospital stay [32,33].

Regarding operative duration, our findings reflect the broader debate in the current literature. While some studies reported slightly shorter operative times for RALP [34,35,36], others demonstrated no meaningful difference [27,37]. This variability is likely driven by the heterogeneity of study populations, particularly with respect to age, weight, and institutional experience [38].

The interpretation of these findings, particularly regarding operative duration, must be viewed through the lens of the surgical learning curve. Our sensitivity analysis revealed that statistical inconsistency persisted even after systematic exclusion of outliers, with heterogeneity for operative time remaining at a high level of 78%. This suggests that the variability was not driven by a single study’s methodology but is rather a reflection of the diverse expertise levels across different pediatric centers. In the context of robotic surgery for infants and neonates, the transition from conventional laparoscopy involves a significant adjustment period [16]. Variations in team synchronization, docking times, and the surgeon’s familiarity with the robotic platform in extremely small operative fields act as primary confounders [38].

Ultimately, this inherent variability in surgical performance, combined with the observational nature of the included studies, is reflected in our formal GRADE assessment. While we identified promising clinical trends, the overall certainty of evidence for operative time was rated as “very low”. However, it is important to balance this rating with the fact that the individual studies demonstrated high methodological quality according to the Newcastle–Ottawa Scale (NOS), representing the best available evidence currently obtainable in this niche infant population.

Cost-effectiveness continues to be debated. Hu et al. reported a threefold increase in hospital costs for RALP [25]. Likewise, randomized data from Silay et al. confirmed the higher cost of robotic surgery, despite its comparable efficacy and safety, than LP [34]. We demonstrated a consistent reduction in hospital stay (MD = −0.73 to −1.69 days), which directly translates to lower per-patient ward costs. In a pediatric setting, shorter hospitalization not only reduces direct hospital expenses but also minimizes the indirect costs associated with parental absence from work and the overall burden on the healthcare system.

### 4.1. Clinical Implications and Future Directions

The demonstrated hospitalization benefit supports RALP as a valuable option for pyeloplasty in young children. However, cost-effectiveness considerations and long-term functional outcomes require further investigation. Future research should prioritize prospective designs, standardized outcome measures, and detailed reporting of robotic platforms and surgeon experience.

### 4.2. Limitations of This Study

This study contains a number of limitations. First, all included studies were retrospective cohorts lacking randomization, which increased the risk of confounding by unmeasured factors such as surgeon experience or institutional protocols. Consequently, according to the GRADE approach, the certainty of evidence for all evaluated outcomes remained at a “very low” level. Second, we observed very serious statistical inconsistency, particularly for operative time and length of hospital stay. Although our leave-one-out sensitivity analysis successfully identified an outlier for hospital stay, the heterogeneity for operative time remained high even after removing outliers. This suggests that factors such as the surgical learning curve and diverse institutional protocols are inherent sources of variability in infant RALP. Third, the total sample size across the five included studies was relatively small, contributing to serious imprecision for several outcomes. This is reflected in the wide confidence intervals for success and complication rates. Additionally, the exploratory subgroup analyses were inherently underpowered due to the limited number of studies, necessitating caution in their interpretation. Fourth, outcome definitions and reporting (e.g., complications, length of stay) varied across studies, introducing potential bias and limiting the precision of pooled estimates. Furthermore, several studies reported summary statistics as medians with ranges or interquartile intervals, requiring conversion to approximate means and standard deviations for pooling. Such transformations can introduce additional imprecision and may have affected the accuracy of the meta-analytic estimates for the continuous outcomes.

## 5. Conclusions

This meta-analysis suggests that RALP in infants and toddlers may be associated with a shorter hospital stay than LP while maintaining comparable safety and efficacy. However, given the high heterogeneity (I2 > 90%) and the very low certainty of evidence, these results must be interpreted with caution. While RALP appears to be a viable option in pediatric surgical armamentarium, the choice of approach should be carefully guided by surgeon expertise, institutional resources, and individual patient factors.

Nonetheless, the current evidence base is limited by small cohort sizes, heterogeneity in patients’ age and weight, and a scarcity of randomized studies. High-quality, multicenter, randomized trials are therefore essential to confirm these observations.

## Figures and Tables

**Table 1:** Characteristics of included studies.

Study	Country	Type	Robotic System Used	Patients RALP + LP = Total	Median Age, Months	Median Weight, kg
Neheman 2018 [14]	Israel	Retrospective	NA	21 + 13 = 34	6.1	7.9
Wong 2021 [15]	China	Retrospective	da Vinci model S	24 + 22 = 46	6	8
Andolfi 2022 [16]	USA	Retrospective	NA	39 + 26 = 65	4 *	NA
Sun 2023 [17]	China	Retrospective	NA	12 + 21 = 33	15.7 *	NA
Bindi 2024 [18]	International	Retrospective	NA	52 + 42 = 94	23.9 *	10.7

* Data presented as mean.

**Table 2:** Summary of the quality of evidence (GRADE) for the main outcomes evaluated.

Outcomes	Anticipated Absolute Effects * (95% CI)		Relative Effect (95% CI)	№ of Participants (Studies)	Certainty of the Evidence (GRADE)
	Risk with LP	Risk with RALP			
Success rate	960 per 1000	983 per 1000 (928 to 996)	OR 2.37 (0.54 to 10.50)	272 (5 non-randomized studies)	⨁◯◯◯ Very low ^a,b,c^
Postoperative complications	161 per 1000	89 per 1000 (44 to 173)	OR 0.51 (0.24 to 1.09)	272 (5 non-randomized studies)	⨁◯◯◯ Very low ^b,c^
Operative time	The mean operative time was 196.62 min	MD: 29.68 min fewer (83.21 fewer to 23.85 more)	-	272 (5 non-randomized studies)	⨁◯◯◯ Very low ^b,c,d^
Time of hospitalization	The mean time of hospitalization was 4.7 days	MD: 1.69 days fewer (2.71 fewer to 0.67 fewer)	-	272 (5 non-randomized studies)	⨁◯◯◯ Very low ^c,e^

^a^ Although definitions of success varied across studies—ranging from isolated ultrasound improvement to comprehensive clinical and functional assessment—the majority of the evidence was based on multidimensional criteria, and no serious indirectness was suspected. ^b^ Downgraded by one level for imprecision: the total sample size (*n* = 272) is below the optimal information size (OIS) threshold, and the 95% confidence interval crosses the line-of-null effect, including both potential benefit and harm. ^c^ Publication bias was rated as undetected. Statistical assessment using funnel plots and formal tests was not performed due to the limited number of studies included for this outcome, as these methods lack sufficient power when fewer than 10 studies are available. ^d^ Downgraded by two levels for very serious inconsistency: statistical heterogeneity was extremely high (I2 = 95%) and the direction of the effect was inconsistent across studies (four studies reported shorter operative times for RALP, while one reported a longer time). ^e^ Downgraded by two levels for very serious inconsistency: statistical heterogeneity was extremely high (I2 = 92%). Although a leave-one-out sensitivity analysis reduced I2 to 19%, the primary pooled estimate remains highly inconsistent due to one outlier study. * Data presented as mean.

## Data Availability

The data presented in this study are available on request from the corresponding author; the data are not publicly available due to privacy.

## Supplementary Materials

The following supporting information can be downloaded at: https://www.mdpi.com/article/10.3390/children13060728/s1, Figure S1: Leave-one-out analysis results [14,15,16,17,18].

## Abbreviations

The following abbreviations are used in this manuscript:

UPJO	Ureteropelvic junction obstruction
RALP	Robotic-assisted laparoscopic pyeloplasty
LP	Laparoscopic pyeloplasty
