# Supplementary material for: Comparative Outcomes of Robot-Assisted and Laparoscopic Pyeloplasty in Infants and Toddlers: A Systematic Review and Meta-Analysis
Source: Children (Basel). 2026 May 23;13(6):728. doi: 10.3390/children13060728 (PMC13296848; doi:10.3390/children13060728)
Supplement: Supplementary file 1 [file children-13-00728-s001.zip › children-4256374-supplementary.pdf]

Supplementary Table S1. Leave-one-out analysis results

Age

		I^2	p-value	MD
Removed author:	none (initial values)	83%	0.11	2.05
Removed author:	Neheman 2018	87%	0.11	2.82
Removed author:	Sun 2023	86%	0.19	1.76
Removed author:	Andolfi 2022	87%	0.09	3.15
**Removed author:**	**Bindi 2024**	**0%**	**0.83**	**0.1**
Removed author:	Wong 2021	86%	0.07	3.3

Weight

		I^2	p-value	MD
Removed author:	none (initial values)	93%	0.87	0.17
Removed author:	Neheman 2018	96%	0.63	0.68
**Removed author:**	**Bindi 2024**	**0%**	**0.02**	**-0.77**
Removed author:	Wong 2021	94%	0.68	0.62

Sex - male

		I^2	p-value	OR
Removed author:	none (initial values)	25%	0.7	0.87
Removed author:	Neheman 2018	0%	0.69	1.13
Removed author:	Andolfi 2022	42%	0.87	0.93
Removed author:	Bindi 2024	17%	0.33	0.64
Removed author:	Wong 2021	43%	0.49	0.73

Laterality - left

		I^2	p-value	OR
Removed author:	none (initial values)	0%	0.35	0.67
Removed author:	Neheman 2018	0%	0.14	0.46
Removed author:	Sun 2023	31%	0.61	0.75
Removed author:	Wong 2021	0%	0.92	0.94

Operative time

		I^2	p-value	MD
Removed author:	none (initial values)	95%	0.28	-29.68
Removed author:	Neheman 2018	96%	0.31	-35.57
Removed author:	Sun 2023	96%	0.41	-28.17
Removed author:	Andolfi 2022	94%	0.11	-44.93
**Removed author:**	**Bindi 2024**	**78%**	**0.68**	**-6.04**
Removed author:	Wong 2021	96%	0.34	-32.83

Postoperative complications

		I^2	p-value	OR
Removed author:	none (initial values)	0%	0.08	0.51
Removed author:	Neheman 2018	0%	0.08	0.46
Removed author:	Sun 2023	0%	0.08	0.51
Removed author:	Andolfi 2022	0%	0.2	0.52
Removed author:	Bindi 2024	0%	0.19	0.58
Removed author:	Wong 2021	0%	0.09	0.48

Hospitalization time

		I^2	p-value	MD
Removed author:	none (initial values)	92%	0.001	-1.69
**Removed author:**	**Neheman 2018**	**19%**	**<0.0001**	**-0.73**
Removed author:	Sun 2023	94%	0.004	-1.68
Removed author:	Andolfi 2022	93%	0.005	-2.13
Removed author:	Bindi 2024	94%	0.008	-2.05
Removed author:	Wong 2021	94%	0.005	-2.02

Success rate

		I^2	p-value	OR
Removed author:	none (initial values)	0%	0.26	2.37
Removed author:	Neheman 2018	0%	0.26	2.7
Removed author:	Sun 2023	0%	0.26	2.37
Removed author:	Andolfi 2022	0%	0.47	2
Removed author:	Bindi 2024	0%	0.26	2.37
Removed author:	Wong 2021	0%	0.35	2.41
